# Change in Diet Quality and Meal Sources during the COVID-19 Pandemic in a Diverse Subset of Men and Women in the Cancer Prevention Study-3

**DOI:** 10.3390/nu15040849

**Published:** 2023-02-07

**Authors:** Caroline Y. Um, Rebecca A. Hodge, Marjorie L. McCullough

**Affiliations:** Department of Population Science, American Cancer Society, Kennesaw, GA 30144, USA

**Keywords:** COVID-19, diet quality, meal source

## Abstract

The COVID-19 pandemic resulted in restrictive measures that caused disruptions in behaviors that may have long-term consequences on diet, health, and chronic disease risk. The aim of this study was to assess longitudinal changes in diet quality from before to during the pandemic among 2335 adult participants (816 males and 1519 females; aged 36–78) of the Cancer Prevention Study-3 cohort. We compared dietary screeners conducted in 2018 and 2020 and calculated a diet quality score, which assigned higher points for recommended foods. Overall diet quality slightly improved among all participants from before to during the pandemic, particularly among males (+0.45 points, *p* < 0.001), White participants (+0.24 points, *p* < 0.001), and participants reporting weight loss (+0.66 points, *p* < 0.001 for 2.25 -< 4.5 kg loss; +1.04 points, *p* < 0.001 for ≥4.5 kg loss); change in diet quality did not differ by other sociodemographic factors. Reported consumption of most food groups decreased, especially whole grains (−0.17 servings/day, *p* < 0.001) and vegetables (−0.21 servings/day, *p* < 0.001), primarily among females, Black participants, and participants who gained ≥2.25 kg. The frequency of meals from outside the home decreased, especially in full-service restaurants (−0.47 times/week, *p* < 0.001) and for ready-to-eat meals (−0.37 times/week, *p* < 0.001). Declines in whole grain and vegetable consumption raise concerns for weight gain in these populations and increased risk of poor metabolic health and chronic disease.

## 1. Introduction

The 2019 coronavirus (COVID-19) pandemic that began in early 2020 has resulted in over 660 million cases and 6.7 million deaths to date worldwide [1]. In the U.S. alone, more than 100 million cases and 1,000,000 deaths occurred by the end of 2022 [2]. The pandemic presented unprecedented challenges not only to workplaces, schools, and various business sectors but also to food systems and food security.

Global lifestyle patterns changed as a result of various pandemic-related factors, including social distancing restrictions to reduce COVID-19 transmission [3,4], disruptions in the food supply [4], higher food prices [5,6], and increased unemployment leading to reduced incomes [6,7]. As a result, studies conducted in the U.S., Croatia, and Belgium reported that common food choice determinants during the pandemic were related to cost, healthfulness of foods to strengthen the immune system, convenience/ease of preparation, mood, and sensory appeal [8,9,10]. In addition, studies conducted in Brazil, Israel, and the United Kingdom found that emotional eating increased in some populations during the pandemic and that it was primarily associated with several negative lifestyle changes, including perceived stress level, depression, sadness, and increased food intake [11,12,13].

Although dietary patterns and diet quality may be expected to substantially change because of these pandemic-related stressors, it is unclear how dietary intakes have actually changed, likely due to differences in study assessment methods and time periods. Various studies from both developed and developing countries reported positive dietary changes among adults, such as increased consumption of fresh produce [14,15,16,17] and decreased consumption of comfort foods [18]. In contrast, decreased consumption of vegetables and fruits [19,20,21,22,23,24] and increased consumption of comfort [25,26,27] and snack foods [15,18,20,27,28,29,30,31,32,33,34] were also reported in many countries.

Though several studies have been conducted in U.S. populations, only one study assessed longitudinal changes in diet, and all study participants resided in one U.S. state [35]. Other studies assessed short-term (2-week) changes in dietary intake [22] or retrospectively assessed perceived dietary changes [15,31,36]; thus, prospective, longitudinal evidence from larger U.S.-based populations is lacking. To address this gap, the present study assessed longitudinal changes in dietary intake, dietary quality, and meal sources from before (2018) to during the COVID-19 pandemic (July/August 2020) among a subgroup of U.S. participants enrolled in the nationwide Cancer Prevention Study-3 (CPS-3) and examined how these dietary changes varied by age, sex, race/ethnicity, income, and changes in body weight.

## 2. Materials and Methods

### 2.1. Study Population

Study participants were a small subset of those enrolled in the CPS-3, a cohort established by the American Cancer Society (ACS) in 2006 to prospectively examine cancer incidence and mortality [37]. For the CPS-3 cohort, over 304,000 cancer-free males and females aged 30 to 65 years were enrolled at in-person ACS fundraising and community events from 2006 and 2013 and completed a baseline survey. Starting in 2015, additional surveys were sent every three years, with a validated semiquantitative food frequency questionnaire (FFQ) [38,39] included in 2015 and 2021 and a brief dietary screener in 2018. The brief screener was included to provide a general measure of diet quality when the FFQ was not administered. It incorporated six items (vegetables, fruits, whole grains, refined grains, red meat, and processed meat) from the National Cancer Institute’s 26-item Dietary Screener Questionnaire (DSQ) that corresponded to ACS dietary recommendations [40] (with modifications to portion size and frequency format to match the CPS-3 design). Additional questions of research interest were included, such as water and alcohol consumption and probiotic use. Participants were asked to report their average consumption over the past year, and frequencies ranged from “never or less than once per month” to “6+ per day.”

In June 2020, an online participant portal was developed and tested in a subgroup of approximately 3000 CPS-3 participants. All participants were invited to take a COVID-19-focused questionnaire (“COVID survey”), which sought to examine how the pandemic affected participants’ dietary habits using the same dietary screener administered in 2018. The survey also investigated the pandemic’s effects on physical and mental health [41,42] and included questions about self-reported weight, physical activity [43], health care access, health insurance, employment status, and financial security. A total of 2429 portal participants completed this survey between July and August 2020, during which time much of the U.S. experienced restrictions on large group gatherings and events but not nationwide lockdowns or curfews, unlike some other countries.

### 2.2. Assessment of Dietary Changes

To assess dietary change, responses to the 2018 dietary screener (reflecting usual intake over the past year) were compared to those collected on the COVID survey in July–August 2020. All line items on the COVID survey were identical to the 2018 survey. Foods included in the questionnaire were vegetables (not including potatoes or iceberg lettuce), fruit (whole, fresh, frozen, canned, or dried), 100% fruit juice, whole grain foods, refined grain foods, red meat, processed meat, sugar-sweetened beverages, and diet beverages. Common portion sizes were specified, e.g., 1 slice of whole grain bread or 2 slices of bacon. The COVID survey had eight possible frequency responses ranging from “Never, or less than once per month” to “4+ per day.” The 2018 survey had nine possible frequencies, with the top two frequencies (“4–5 per day” and “6+ per day”) combined to match the COVID survey response options.

The relative validity of the dietary screener included in the COVID survey was assessed by comparing estimated food group intakes (servings/day) with the validated 2021 FFQ, which asked about dietary habits over the past year (and thus included the COVID survey period). We assessed the correlation in food group intakes (servings/day) between the two dietary assessment methods using Spearman rank correlation coefficients. To assess the presence, direction, and extent of bias in absolute intakes, we calculated the mean differences between food group intakes and Bland–Altman 95% limits of agreement [44]. Of the 2429 participants who completed the COVID survey, 2071 participants also completed the 2021 FFQ and were included in this validation analysis. The correlations were as follows: 0.34 for refined grains (including sweets and desserts), 0.40 for processed meats, 0.46 for whole grains, 0.46 for vegetables, 0.47 for red meats, 0.52 for fruits, 0.54 for fruit juices, 0.62 for sugar-sweetened beverages, and 0.76 for diet beverages. In general, all food groups, except sugar-sweetened beverages, were likely overreported on the FFQ relative to the COVID survey (Table S1) because the FFQ assessed the intake of multiple individual foods within each food group, whereas the COVID survey assessed total food group consumption.

To assess overall diet quality in 2018 and 2020, a score was calculated by assigning points to categories of intake of each food group. Higher points (0–3) were assigned for higher reported intakes (≤1–3 times/month, 1–6 times/week, once/day, and ≥2–3 times/day) of foods recommended by cancer prevention guidelines (vegetables, fruits, and whole grains) [45], while lower points (3–0) were given for higher intakes (≤1–3 times/month, once/week, 2–6 times/week, and ≥once/day) of foods that are not recommended (refined grains and red and processed meat). Sugar-sweetened and diet beverages and fruit juices were each assigned one point for no consumption or <once/month and zero points for any consumption ≥once/month. The total score ranged from 0 to 21 points, with higher scores indicating higher diet quality.

Both the 2018 and COVID surveys also queried the frequency of consuming meals prepared outside the home (e.g., full-service restaurants, fast food restaurants, and store-prepared ready-to-eat meals) and cooked at home. The COVID survey had six possible frequency responses ranging from “Never” to “5+ times per week.” The 2018 survey had seven possible responses, of which the top two frequencies (“5–7 times per week” and “8+ per week”) were combined to match the COVID survey. Lastly, the COVID survey assessed food insecurity using a 2-item Hunger Vital Sign screener [46] and assessed perceptions of changes in consumption of 13 categories of foods and beverages as a result of the pandemic (e.g., “increased,” “decreased,” and “no change”). These items largely overlapped with the dietary screener, except it also included additional questions on sweets, snack foods, dairy, and fish.

Of the 2429 participants who completed the COVID survey, participants were excluded if they were missing the dietary screener in 2018 (N = 3) or 2020 (N = 62), reported only consuming beverages but no food items in 2018 (N = 1) or 2020 (N = 2), did not answer the questions about perceived dietary changes during the pandemic (N = 2), or were missing body mass index (BMI) in 2018 (N = 4) or 2020 (N = 20). The final analytic cohort included 2335 participants (816 males and 1519 females).

### 2.3. Statistical Analysis

Within-participant differences in reported food group intakes and diet quality scores from 2018 to the 2020 COVID survey were calculated and then averaged. Change in the frequency of meals consumed from different sources (home-cooked or prepared outside the home) from 2018 to 2020 was also calculated. Paired *t*-tests were used to evaluate the significance of the changes in dietary behaviors from 2018 to 2020.

Changes in diet and meal sources were also examined stratified by sex, age (<60 and ≥60 years), race/ethnicity (White, Black, Latino/a, Asian/Pacific Islander, and American Indian/Alaskan Native), education (less than college graduate and college graduate or higher), income (<$50,000; $50,000–$99,999; $100,000–$149,999; and ≥$150,000) and weight change (calculated using self-reported weight in 2018 and 2020; categorized as lost/gained ≥4.5 kg, lost/gained 2.25 -<4.5 kg, and lost/gained <2.25 kg). Weight change categories were based on the interquartile range (−2.25 to +2.25 kg) and 10th and 90th percentiles (−4.5 and +4.5 kg, respectively). All stratification variables were assessed at study enrollment (sex, race/ethnicity, and education) or in 2018 (age and income); only weight change was calculated using data collected at both time points. Observed differences between strata were examined by unpaired *t*-test or ANOVA. All analyses were conducted in R (version 4.0.2).

## 3. Results

### 3.1. Participants’ Sociodemographic Characteristics

In 2020, 2335 participants completed the COVID survey who had a mean age of 56 years and were primarily female (65%), non-Latino White (76%), and college-educated (78% college graduate or higher). The mean diet quality score was 12.4 (SD 3.6, range 1–21). Food insecurity was uncommon in this population, with less than 5% of participants reporting being worried that food supplies would run out (4.6%) or that food would not last until their next paycheck (2.2%).

### 3.2. Change in Diet Quality

Characteristics of the study population by a 2-year change in the diet quality score are presented in Table 1. Overall, there were no differences in most factors between participants who improved, declined, or did not change their diet quality score from 2018 to 2020. White, Asian/Pacific Islander, and Black participants were more likely to have improved diet quality, while more Latino/a and American Indian/Alaskan Native participants had decreased diet quality, although these differences were not statistically significant. Participants who reported weight loss since 2018 (*p* < 0.001) and participants who had a history of cancer prior to 2018 (*p* = 0.05) were also more likely to have improved diet quality during this period. Overall, fast food consumption decreased among participants but decreased the least among participants whose diet quality declined. The frequency of home cooking decreased the most among those participants whose diet quality did not change or declined.

The average diet quality score improved from 2018 to 2020 (0.20 points, *p* < 0.001) among all participants, which was driven by small, but statistically significant, improvements in the component scores for refined grains, red meat, and all beverages (Table 2). These improvements in component scores reflect improved diet quality due to decreased consumption of these food groups, except for diet beverages, which did not change. Conversely, decreased consumption of vegetables, fruits, and whole grains led to a decrease in points from these food groups (reflecting poorer diet quality), though the point change for fruits was not statistically significant (*p* = 0.10). The greatest decrease in consumption was observed for vegetables (−0.21 servings/day, *p* < 0.001) and whole grains (−0.17 servings/day, *p* < 0.001).

#### Change in Diet Quality among Subgroups

**Figure 1:**
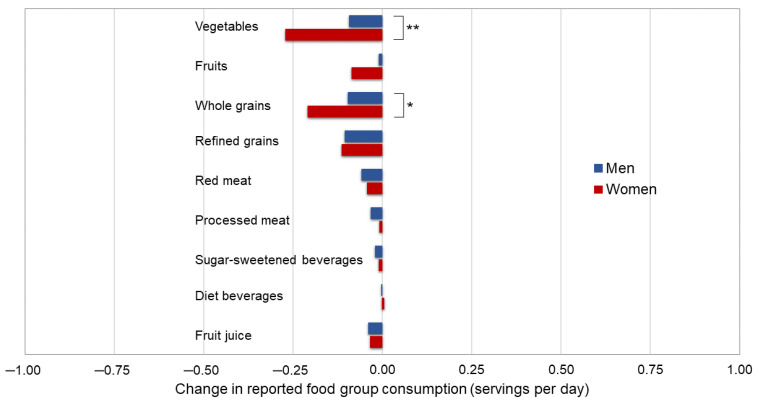
Mean change in individual food group consumption (servings per day) from before (2018) to during (July/Aug 2020) the COVID-19 pandemic among a subgroup of Cancer Prevention Study-3 cohort participants, stratified by sex (N = 2335). *p*-values between strata were examined by *t*-tests and are presented using an asterisk for *p* < 0.05 and two asterisks for *p* < 0.001.

The change in diet quality score was statistically significantly different between males and females (P_interaction_ = 0.001), with improved average scores among males (0.45 points, *p* < 0.001) but not among females (0.07 points, *p* = 0.32). Females had a greater decreased intake of vegetables (−0.27 servings/day, *p* < 0.001; P_interaction_ < 0.001 by sex) and whole grains (−0.21 servings/day, *p* < 0.001; P_interaction_ = 0.01 by sex) compared to males, but changes in other food groups did not statistically significantly differ by sex (Figure 1 and Table S2). Change in diet quality score and component food groups did not vary by age (<60 and ≥60 years), education (<4-year college, college graduate, and graduate school), or income (<$50,000; $50,000–$99,999; $100,000–$149,999; and ≥$150,000) in this younger, mostly educated and employed population (data not shown).

There were no statistically significant differences in changes in overall diet quality scores across racial/ethnic groups (*p* = 0.58), which significantly improved only among White participants (0.24 points, *p* < 0.001; Table S3). There was a statistically significant decrease in consumption of most food groups among White participants, while vegetable intake decreased the most among Black participants (−0.40 servings/day, *p* = 0.03). Latino/a participants also decreased their intake of vegetables (*p* = 0.02), in addition to whole (*p* < 0.001) and refined (*p* = 0.01) grains, while Asian/Pacific Islander participants decreased their intake of sugar-sweetened beverages (*p* = 0.03). There were no statistically significant changes in consumption among American Indian/Alaskan Native participants.

**Figure 2:**
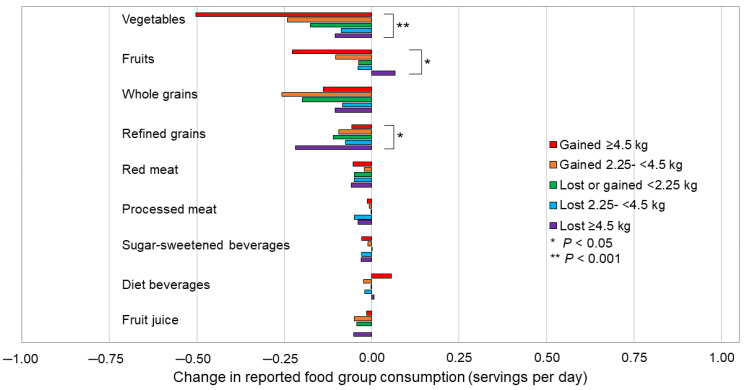
Mean change in individual food group consumption (servings per day) from before (2018) to during (July/Aug 2020) the COVID-19 pandemic among a subgroup of Cancer Prevention Study-3 cohort participants, stratified by weight change categories (N = 2335). *p*-values between strata were examined by ANOVA and are presented using an asterisk for *p* < 0.05 and two asterisks for *p* < 0.001.

Change in diet quality score from 2018 to 2020 significantly varied by categories of weight change (*p* < 0.001; Table S4). Overall, participants who gained at least 2.25 kg had a decline in diet quality score, though the change was statistically significant only among participants who gained ≥4.5 kg (−0.40, *p* = 0.01). While all groups decreased consumption of vegetables, only participants who experienced minimal weight change (lost or gained <2.25 kg) or gained at least 2.25 kg significantly decreased intake (Figure 2 and Table S4). Change in fruit intake also significantly differed across weight change groups, with only participants who lost ≥4.5 kg reporting increased intake (0.07 servings/day, *p* = 0.27). All groups also decreased consumption of refined grains, but the greatest decline was reported among participants who lost ≥4.5 kg (−0.22 servings/day, *p* < 0.0001).

### 3.3. Perceived Change in Dietary Intake

Most participants did not perceive any changes in their dietary intake due to the COVID-19 pandemic (Figure S1). Among participants who perceived any change, more participants perceived an increase, rather than a decrease, in consumption of most foods, except for pizza, processed meats, frozen meals, red meat, and sugar-sweetened beverages, for which decreased intakes were perceived by a greater proportion of participants.

### 3.4. Change in Meal Source

Reported frequency of meals from all sources (Table S5) decreased among participants (*p* = 0.002 for home-cooked meals; *p* < 0.001 for other sources), with the greatest decreases observed in meals from full-service restaurants and prepared ready-to-eat meals (−0.47 and −0.37 times per week, respectively). These changes were mostly consistent across sex and racial/ethnic groups, although the frequency of cooking at home statistically significantly decreased only among males (*p* = 0.003) and White participants (*p* < 0.001). Additionally, the frequency of meals from fast food restaurants did not statistically significantly change among Asian/Pacific Islander and American Indian/Alaskan Native participants, and the frequency of meals from full-service restaurants did not statistically significantly change among Black and American Indian/Alaskan Native participants.

## 4. Discussion

The findings from this longitudinal study of U.S. adults suggest that dietary changes during the COVID-19 pandemic varied by population characteristics. Overall diet quality improved among males, White participants, and participants who lost weight from 2018 to July/August 2020, while the greatest decreases in consumption of healthy foods, particularly vegetables and whole grains, occurred in females, Black participants, and participants who gained weight. The frequency of meals from outside the home decreased among most groups, while the frequency of home-cooked meals only decreased among males and White participants.

Consumption of most food groups decreased from before to during the COVID-19 pandemic among all participants in this study, but the diet quality score slightly improved only among certain groups. This improvement in the diet quality score was likely driven by a greater decrease in the consumption of unhealthy foods compared to healthy foods. However, groups that did not improve their diet quality score (females, non-White participants, and participants who gained at least 2.25 kg) experienced greater decreases in healthy foods (vegetables, fruits, and whole grains) in comparison to unhealthy foods, which is concerning. Vegetables and whole grains are key contributors of insoluble and soluble dietary fiber, nutrients, and phytochemicals, which are associated with lower risk of cardiovascular and coronary heart disease [47,48], type 2 diabetes [49], and certain cancers, including colorectal [50]. Therefore, these shifts in dietary intakes during the pandemic may increase the risk of chronic diseases, especially if sustained long-term. Additional research is needed to identify the specific drivers of decreases in vegetable and whole grain consumption reported herein.

Various international studies that assessed the impact of the pandemic on dietary behaviors reported both favorable and unfavorable changes, particularly in vegetable and fruit intakes [51,52]. One study that also assessed longitudinal changes in whole and refined grain consumption among Canadian adults reported increased consumption of whole grains, which contrasts with our findings [53]. This study, which utilized online 24 h dietary recalls administered before and during the beginning (April/May 2020) of the COVID-19 pandemic, also reported improvements in overall diet quality assessed using the Healthy Eating Index (HEI) 2015. Overall improvements in diet quality in the Canadian study were attributable to increased consumption of whole grains, greens and beans, vegetables, dairy, and total proteins and decreased consumption of refined grains and added sugars.

Two additional studies [54,55] similarly assessed changes in adherence to recommended dietary patterns. In a study of Croatian adults (N = 4281) who reported their perceived dietary changes during the early months of the pandemic, higher perceived adherence to the Mediterranean diet was reported among participants who were female, more educated, between 18 and 50 years old, and had normal BMI [55]. In contrast, French adults (N = 938) who retrospectively reported their dietary behaviors for the month before and during the first month of lockdown restrictions reported decreased adherence to French dietary recommendations due to greater increases in processed meat, sweet-tasting beverages, and alcohol consumption despite increases in fruits, vegetables, pulses, fish, and seafood consumption [54]. We did not observe statistically significant differences in diet quality by age, BMI, education, or income, but given the varying results among the current findings and previous studies, further understanding of factors that may be associated with changes in diet quality during the pandemic is needed.

Few additional studies examined longitudinal changes in dietary intake from before to during the pandemic. A study conducted in the U.S. by Gibbs et al. found no changes in diet from before to after the pandemic except for decreased consumption of red meat [35]. Diet was assessed in 2018 and from May to June 2020, which was similar to our study period; however, the study population was limited to desk workers residing in Pennsylvania. An additional study by Mitchell et al. assessed dietary intakes among users of the weight loss app “Noom” (Noom Inc, New York, NY, USA) from before to after the pandemic began, but the time period was limited to one week before and the first week of the COVID-19 lockdown [22]. This study similarly reported decreased consumption of all food groups and a greater decrease in vegetable consumption among females. However, given the short study time period and that study participants were users of a weight loss application, it is unclear if these dietary changes occurred as a result of the pandemic. Neither of the abovementioned studies reported results by race/ethnicity due to the lack of diversity or racial/ethnic data.

Additional studies conducted in the U.S. retrospectively assessed perceived dietary changes [15,31,36]. Though these studies utilized online surveys that asked participants to report how their diet changed from before the pandemic to the current time period (ranging from April to May 2020), different methods of dietary assessment were used. Participants who were asked about perceived changes in their consumption of various foods and beverages reported increased consumption of almost all items, including fruits, vegetables, processed foods, sugar-sweetened beverages, and snack foods [15]. In our study, although consumption of most food groups decreased, most study participants did not perceive a change in their consumption. For food groups that were assessed for both perceived and documented intakes, people who perceived any change generally perceived increased intake of fruits, vegetables, and whole grains despite decreased self-reported intake from 2018 to 2020. These differences in perceived vs. documented changes may be due to various factors. First is the small magnitude of documented changes that occurred from before to during the pandemic (<½ serving/day) since dietary changes may only be perceived with large or healthier shifts in eating patterns [56]. Studies also suggest that although, on average, perceived diet quality tends to be healthier than measured diet quality [57,58], individuals with high perceived diet quality are not necessarily meeting definitions of various healthy dietary patterns [58]. Lastly, perceived changes in dietary patterns may be influenced by other factors, such as the sight and smell of foods, cravings, boredom, and stress [31]. In a retrospective assessment of perceived dietary behaviors and stress conducted in April 2020, American participants reported higher levels of fasting, restricting eating, and skipping meals as well as overeating, but these behaviors were also correlated with higher stress scores [36].

Most participants in our study reported decreased consumption of meals from outside of the home, as well as cooking at home, during the pandemic, though the frequency of cooking at home did not change among females or non-White racial/ethnic groups. Given the restrictions enforced during the COVID-19 pandemic, it would be expected that participants may have cooked at home more often, as reported in U.S. population surveys [35,59,60,61]. Though home cooking may influence adherence to healthier dietary patterns [55,62], we observed that participants who improved diet quality scores did not change their frequency of home cooking (mean 0.001 (SD 1.7) times/wk), while participants who decreased diet quality also decreased their frequency of home cooking (mean −0.2 (SD 1.7) times/wk). However, the frequency of home cooking also decreased among White participants who improved diet quality. One potential explanation may be that the highest frequency option on both the 2018 and COVID surveys for cooking at home was “≥5 times per week”, so changes in frequency greater than this amount (e.g., decrease from 14 to 7 times per week) may have been missed. Reported consumption of meals from outside the home decreased in this study, while a U.S. food and beverage marketing communications firm reported an increase in take-out and delivery meal consumption from April to December 2020 [60]. This discrepancy may relate to the timing of these assessments, as participant comfort level with eating out or purchasing take-out food may have increased during the pandemic but decreased in comparison to prepandemic levels.

This study is not without limitations. One limitation is the representativeness of our study population in comparison to the U.S. population. In this study, 35% of participants were male, which is higher than the overall CPS-3 cohort (23%) [37] but lower than the U.S. population (49.5%) [63]. In addition, 76.1% of study participants were non-Latino/a White, which is more representative of the U.S. population (75.8% White) than the overall CPS-3 cohort (83% White). However, our study participants were more highly educated (37% college graduates) and of higher socioeconomic status (28.1% reported a 2018 household income of $100,000–$149,999) compared to the U.S. population (23% college graduates and 15.6% reported a 2018 household income of $100,000–$149,999) [64]. In addition, a smaller proportion of our study population (less than 5%) reported food insecurity compared to the U.S. population (10.2%) [65].

We also recognize that the association of diet and weight changes cannot be considered causal as they were measured concurrently; further, we were unable to determine if weight changes were intentional. Weight was also self-reported, so there may be inaccuracy in the calculated changes in weight, though the high validity of self-reported weight with objectively measured weight in CPS-3 participants has already been demonstrated [66]. Lastly, we recognize that, for most groups, the reported dietary changes were relatively small and may not be clinically meaningful. However, additional studies are needed to understand whether the magnitude of these dietary changes remained stable or continued to change during the pandemic. Strengths of this study included its large sample size, in comparison to most studies conducted in the U.S., with sufficient numbers and range of exposures to detect dietary changes within several racial/ethnic and demographic subgroups. Another strength of this study was the ability to compare perceived dietary changes with reported dietary changes from before to during the pandemic. Additionally, we assessed postpandemic diet in July/August 2020, which was later than previous studies, so many Americans may have better adjusted to restrictions and changes related to the pandemic. However, additional studies are needed to increase understanding of longer-term dietary changes related to the pandemic.

## 5. Conclusions

In summary, while we observed an overall slight increase in diet quality from before to during the COVID-19 pandemic, unhealthy changes in dietary behaviors were also observed even among this well-educated, higher-socioeconomic-status sample of American adults. Although many restrictions have now been lifted across the world, surges in variants may continue to interrupt dietary behaviors, and declines in vegetable and whole grain consumption could persist and contribute to unhealthier dietary patterns, especially among females and certain racial/ethnic populations. The persistence or further exacerbation of these unhealthy dietary patterns may increase the risk of weight gain and chronic disease and potentially widen existing disparities in obesity and chronic disease risk. Specific public health efforts may be needed in certain communities to identify and reduce barriers to healthy foods so that populations are not disproportionately affected by future pandemics that impact global food systems.

## Figures and Tables

**Table 1:** Characteristics of Cancer Prevention Study-3 participants who completed an online COVID-19 questionnaire in July/August 2020 (N = 2335), overall and by two-year change in diet quality score.

	Overall	Change in Diet Quality Score ^1^			
		Improved	No Change	Declined	*p* ^3^
	N = 2335 ^2^	N = 1041 ^2^	N = 382 ^2^	N = 912 ^2^	
Change in diet score	0.2 (2.8)	2.6 (1.7)	0.0 (0.0)	−2.4 (1.6)	<0.001
Range	−10 to 13	1 to 13	0	−10 to −1	
Age, years	55.6 (9.7)	55.8 (9.5)	56.1 (9.6)	55.1 (9.9)	0.20
Sex					0.12
Male	816	385 (47%)	134 (16%)	297 (36%)	
Female	1519	656 (43%)	248 (16%)	615 (40%)	
Race/Ethnicity					0.18
White	1777	806 (45%)	294 (17%)	677 (38%)	
Latino/a	298	118 (40%)	50 (17%)	130 (44%)	
Asian/Pacific Islander	88	40 (45%)	12 (14%)	36 (41%)	
Black	81	43 (53%)	7 (8.6%)	31 (38%)	
American Indian/Alaskan Native	44	13 (30%)	9 (20%)	22 (50%)	
Other/missing	47	21 (45%)	10 (21%)	16 (34%)	
Education					0.38
<4-year college	516	229 (44%)	76 (15%)	211 (41%)	
College graduate	872	406 (47%)	137 (16%)	329 (38%)	
Graduate school	947	406 (43%)	169 (18%)	372 (39%)	
Household income in 2018					0.76
<$50,000	228	105 (46%)	35 (15%)	88 (39%)	
$50,000–$99,999	701	319 (46%)	110 (16%)	272 (39%)	
$100,000–$149,999	656	291 (44%)	100 (15%)	265 (40%)	
≥$150,000	750	326 (43%)	137 (18%)	287 (38%)	
Smoking status in 2020					0.48
Never	1678	751 (45%)	268 (16%)	659 (39%)	
Current	31	12 (39%)	3 (9.7%)	16 (52%)	
Former	626	278 (44%)	111 (18%)	237 (38%)	
Alcohol in 2020, g/day	0.6 (0.8)	0.6 (0.8)	0.6 (0.8)	0.6 (0.8)	0.97
Physical activity, total MET-hrs/week	7.2 (6.5)	7.2 (6.5)	7.1 (6.1)	7.2 (6.8)	0.90
Body mass index in 2018, kg/m^2^	27.5 (6.0)	27.5 (5.7)	27.5 (6.4)	27.5 (6.1)	0.53
Body mass index in 2020, kg/m^2^	27.5 (6.1)	27.3 (5.8)	27.4 (6.4)	27.8 (6.3)	0.24
Change in weight, kg					<0.001
Lost ≥ 4.5 kg	333	180 (54%)	57 (17%)	96 (29%)	
Lost 2.25 -<4.5 kg	321	168 (52%)	53 (17%)	100 (31%)	
Lost or gained < 2.25 kg	1029	437 (42%)	180 (17%)	412 (40%)	
Gained 2.25 -<4.5 kg	320	126 (39%)	42 (13%)	152 (48%)	
Gained ≥ 4.5 kg	332	130 (39%)	50 (15%)	152 (46%)	
Self-reported COVID-19 infection ^4^	58	22 (38%)	8 (14%)	28 (48%)	0.35
Risk of COVID-19 complications ^5^					0.42
Low	645	274 (42%)	110 (17%)	261 (40%)	
Medium	850	401 (47%)	134 (16%)	315 (37%)	
High	840	366 (44%)	138 (16%)	336 (40%)	
History of cancer	124	62 (50%)	26 (21%)	36 (29%)	0.05
Marital status in 2018					0.18
Married/w partner	1858	830 (45%)	306 (16%)	722 (39%)	
Never married	173	81 (47%)	18 (10%)	74 (43%)	
Divorced, separated, or widowed	304	130 (43%)	58 (19%)	116 (38%)	
Living situation during pandemic					0.69
Lives alone	278	119 (43%)	50 (18%)	109 (39%)	
Lives with other people	2057	922 (45%)	332 (16%)	803 (39%)	
Number of people in home	2.4 (1.4)	2.4 (1.4)	2.4 (1.5)	2.3 (1.3)	0.60
Caregiver for other adults or children in home	534	231 (43%)	94 (18%)	209 (39%)	0.60
Change in meal sources (times/week)					
Fast food	−0.1 (0.9)	−0.2 (1.0)	−0.2 (0.8)	−0.03 (0.8)	<0.001
Full service	−0.5 (1.2)	−0.5 (1.2)	−0.4 (1.2)	−0.4 (1.0)	0.54
Prepared	−0.7 (1.1)	−0.4 (1.1)	−0.3 (1.1)	−0.3 (1.0)	0.09
Home cooked	−0.1 (1.7)	0.001 (1.7)	−0.2 (1.7)	−0.2 (1.7)	0.01

Abbreviations: COVID-19, coronavirus disease 2019; MET, metabolic equivalent of task. ^1^ Diet quality score calculated as 0–3 points for higher intake (≤1–3 times/month, 1–6 times/week, once/day, and ≥2–3 times/day) for vegetables, fruits, and whole grains; 0–3 points for lower intake (≤1–3 times/month, once/week, 2–6 times/week, and ≥once/day) of refined grains, red meat, and processed meat; 1/0 points for no or <once/month/any consumption of sugar-sweetened beverages, diet beverages, or fruit juices. Change in diet score was calculated by subtracting 2018 score from 2020 score. ^2^ Values shown in each column are means ± standard deviation or N (%). ^3^ *p*-values calculated using Kruskal–Wallis rank sum test or Pearson’s chi-squared test. ^4^ Self-reported COVID-19 infection includes participants who tested positive for COVID-19 or were suspected of having COVID-19 by their physician. ^5^ Participants were considered to be at high risk of COVID-19 complications if they were obese, current smokers, or had a history of heart attack, stroke, emphysema, type II diabetes, kidney disease, cancer, or heart bypass surgery. Participants were considered to be at medium risk if they were overweight or had HIV, asthma, type I diabetes, high blood pressure, or chronic liver disease. Participants were considered to be at low risk if they had none of the previous conditions.

**Table 2:** Mean change in diet quality score (points) and individual food group consumption (points and servings per day) from before (2018) to during (July/Aug 2020) the COVID-19 pandemic among a subgroup of Cancer Prevention Study-3 cohort participants (N = 2335).

	Points ^1^				Servings Per Day			
	2018 ^2^	2020 ^2^	Mean Change ^2^	*p* ^3^	2018 ^4^	2020 ^4^	Mean Change ^4^	*p* ^3^
Diet quality score ^5^	12.24 ± 3.63	12.45 ± 3.57	0.20	<0.001	--	--	--	--
Vegetables	2.08 ± 0.96	1.99 ± 0.95	−0.09	<0.001	1.79 ± 1.35	1.58 ± 1.23	−0.21	<0.001
Fruits	1.95 ± 0.97	1.92 ± 0.97	−0.03	0.10	1.50 ± 1.20	1.45 ± 1.15	−0.06	0.01
Whole grains	1.45 ± 0.97	1.29 ± 0.94	−0.16	<0.001	0.98 ± 1.03	0.81 ± 0.91	−0.17	<0.001
Refined grains	1.40 ± 1.06	1.55 ± 1.05	0.15	<0.001	0.59 ± 0.72	0.48 ± 0.60	−0.11	<0.001
Red meat	1.55 ± 0.94	1.65 ± 0.95	0.10	<0.001	0.41 ± 0.46	0.37 ± 0.41	−0.05	<0.001
Processed meat	2.16 ± 0.94	2.18 ± 0.92	0.03	0.12	0.23 ± 0.34	0.21 ± 0.30	−0.02	0.03
Sugar-sweetened beverages	0.55 ± 0.50	0.65 ± 0.48	0.09	<0.001	0.16 ± 0.45	0.14 ± 0.43	−0.01	0.17
Diet beverages	0.59 ± 0.49	0.61 ± 0.49	0.02	0.04	0.34 ± 0.81	0.34 ± 0.77	0.003	0.83
Fruit juice	0.52 ± 0.50	0.61 ± 0.49	0.09	<0.001	0.22 ± 0.46	0.18 ± 0.41	−0.04	<0.001

^1^ Points for the diet quality score calculated as 0–3 points for higher intake (≤1–3 times/month, 1–6 times/week, once/day, and ≥2–3 times/day) for vegetables, fruits, and whole grains; 0–3 points for lower intake (≤1–3 times/month, once/week, 2–6 times/week, and ≥once/day) of refined grains, red meat, and processed meat; 1/0 points for no or <once/month/any consumption of sugar-sweetened beverages, diet beverages, or fruit juices. ^2^ Values for 2018 and 2020 are mean ± SD expressed as points. Mean change calculated as the difference between 2018 and 2020, expressed as points for the diet quality score and individual food groups. ^3^ *p*-values calculated using paired *t*-tests. ^4^ Values for 2018 and 2020 are mean ± SD expressed as servings per day. Mean change calculated as the difference between 2018 and 2020, expressed as servings per day for individual food groups. ^5^ Diet quality score calculated as the sum of component food groups.

## Data Availability

The data generated in this study are available upon reasonable request from the corresponding author. The Cancer Prevention Study (CPS) data access policies and procedures are publicly available here: https://www.cancer.org/content/dam/cancer-org/research/epidemiology/cancer-prevention-study-data-access-policies.pdf (accessed on 11 January 2023).

## Supplementary Materials

The following supporting information can be downloaded at: https://www.mdpi.com/article/10.3390/nu15040849/s1, Figure S1: Perceived changes in food group consumption during the COVID-19 pandemic among a subgroup of Cancer Prevention Study-3 participants (N = 2335); Table S1: Average mean differences in intake and Bland–Altman limits of agreement (LOA) between food group intakes (servings per day) estimated by the 2020 COVID-19 brief dietary screener and 2021 Cancer Prevention Study-3 (CPS-3) food frequency questionnaire among a subgroup of CPS-3 cohort participants.; Table S2. Mean change in diet quality score (points) and individual food group consumption (servings per day) from before (2018) to during (July/Aug 2020) the COVID-19 pandemic among a subgroup of Cancer Prevention Study-3 cohort participants, stratified by sex (N = 2335). Table S3: Mean change in diet quality score (points) and individual food group consumption (servings per day) from before (2018) to during (July/Aug 2020) the COVID-19 pandemic among a subgroup of Cancer Prevention Study-3 cohort participants, stratified by race/ethnicity (N = 2335); Table S4: Mean change in diet quality score (points) and individual food group consumption (servings per day) from before (2018) to during (July/Aug 2020) the COVID-19 pandemic among a subgroup of Cancer Prevention Study-3 cohort participants, stratified by weight change categories during this period (N = 2335); Table S5: Reported frequency of and mean change in meal sources (times per week) from before (2018) to during (July/Aug 2020) the COVID-19 pandemic among a subgroup of Cancer Prevention Study-3 participants, stratified by sex and race/ethnicity (N = 2335).
