# Supplementary material for: Change in Diet Quality and Meal Sources during the COVID-19 Pandemic in a Diverse Subset of Men and Women in the Cancer Prevention Study-3
Source: Nutrients. 2023 Feb 7;15(4):849. doi: 10.3390/nu15040849 (PMC9962248; doi:10.3390/nu15040849)

Table S1. Average mean differences in intake and Bland-Altman limits of agreement (LOA) between food group intakes (servings per day) estimated by the 2020 COVID-19 brief dietary screener and 2021 Cancer Prevention Study-3 (CPS-3) food frequency questionnaire among a subgroup of CPS-3 cohort participants.

Food group	Overall (N = 2071) [1]		Men (N = 721)		Women (N = 1350)	
	Mean difference [2] (servings/day)	95% LOA	Mean difference [2] (servings/day)	95% LOA	Mean difference [2] (servings/day)	95% LOA
Vegetable	−1.65	−5.29, 2.00	−1.65	−4.97, 1.67	−1.64	−5.45, 2.16
Fruit	−0.81	−3.59, 1.97	−0.96	−3.72, 1.81	−0.73	−3.51, 2.05
Whole grain	−0.40	−2.30, 1.50	−0.50	−2.55, 1.55	−0.35	−2.16, 1.46
Refined grain	−0.89	−2.75, 0.98	−1.08	−3.10, 0.95	−0.78	−2.53, 0.96
Red meat	−0.02	−0.78, 0.74	−0.01	−0.85, 0.82	−0.03	−0.75, 0.70
Processed meat	−0.08	−0.82, 0.65	−0.13	−1.11, 0.84	−0.06	−0.61, 0.49
Sugar-sweetened beverages	0.05	−0.60, 0.70	0.05	−0.63, 0.72	0.05	−0.59, 0.70
Diet beverages	−0.07	−1.18, 1.04	−0.08	−1.09, 0.93	−0.07	−1.23, 1.09
Fruit juice	−0.04	−0.87, 0.78	−0.06	−0.98, 0.87	−0.04	−0.80, 0.73

Abbreviation: LOA, limits of agreement

[1] There were 2071 study participants who completed both the 2020 COVID-19 brief dietary screener and the 2021 food frequency questionnaire.

[2] Mean difference calculated as the average difference in intake (servings per day) estimated from the 2020 COVID-19 brief dietary screener and the 2021 Cancer Prevention Study-3 food frequency questionnaire.

Supplementary Files

Table S2. Mean change in diet quality score (points) and individual food group consumption (servings per day) from before (2018) to during (July/Aug 2020) the COVID-19 pandemic among a subgroup of Cancer Prevention Study-3 cohort participants, stratified by sex (N = 2335).

	Male (N = 816)		Female (N = 1519)	
	Mean Change [1]	*P* [2]	Mean Change [1]	*P* [2]
Diet quality score[4] (points)	0.45	<0.001	0.07	0.32
Food groups (servings/day)				
Vegetables	−0.09	0.02	−0.27	<0.001
Fruits	−0.01	0.78	−0.09	0.01
Whole grains	−0.10	0.003	−0.21	<0.001
Refined grains	−0.11	<0.001	−0.11	<0.001
Red meat	−0.06	<0.001	−0.04	<0.001
Processed meat	−0.03	0.03	−0.01	0.36
Sugar-sweetened beverages	−0.02	0.25	−0.01	0.40
Diet beverages	−0.001	0.97	0.01	0.77
Fruit juice	−0.04	0.02	−0.03	<0.001

[1] Mean change (difference between 2018 and 2020) expressed as points for the diet quality score and servings per day for individual food groups.
[2] *p*-values calculated using paired *t*-tests.
[3] *p*-values by race/ethnicity calculated using analysis of variances (ANOVA).
[4] Diet quality score calculated as 0-3 points for higher intake (≤1-3 times/month, 1-6 times/week, once/day, ≥2-3 times/day) for vegetables, fruits, whole grains; 0-3 points for lower intake (≤1-3 times/month, once/week, 2-6 times/week, ≥once/day) of refined grains, red meat, processed meat; 1/0 points for no or <once/month/any consumption of sugar-sweetened beverages, diet beverages, or fruit juices. Change in diet score was calculated by subtracting 2018 score from 2020 score.

Table S3. Mean change in diet quality score (points) and individual food group consumption (servings per day) from before (2018) to during (July/Aug 2020) the COVID-19 pandemic among a subgroup of Cancer Prevention Study-3 cohort participants, stratified by race/ethnicity (N = 2335).

	White (N = 1777)		Black (N = 81)		Latino/a (N = 298)		Asian/ Pacific Islander (N = 88)		American Indian/ Alaskan Native (N = 44)		*P* by race/ethnicity[3]
	Mean Change [1]	*P* [2]	Mean Change [1]	*P* [2]	Mean Change [1]	*P* [2]	Mean Change [1]	*P* [2]	Mean Change [1]	*P* [2]	
Diet quality score[4] (points)	0.24	<0.001	0.38	0.34	−0.03	0.87	0.08	0.77	−0.09	0.83	0.58
<u>Food groups (servings/day)</u>											
Vegetables	−0.22	<0.001	−0.40	0.03	−0.16	0.02	−0.12	0.43	−0.09	0.71	0.61
Fruits	−0.08	0.002	−0.02	0.89	−0.07	0.30	0.23	0.10	0.22	0.10	0.11
Whole grains	−0.16	<0.001	−0.13	0.22	−0.20	<0.001	−0.17	0.07	−0.17	0.31	0.98
Refined grains	−0.12	<0.001	−0.05	0.46	−0.10	0.01	−0.04	0.72	−0.09	0.56	0.87
Red meat	−0.05	<0.001	−0.09	0.10	−0.04	0.12	0.00	0.99	−0.16	0.25	0.42
Processed meat	−0.02	0.03	−0.08	0.23	−0.01	0.80	0.02	0.43	0.01	0.93	0.64
Sugar-sweetened beverages	−0.01	0.49	0.00	0.93	0.00	0.89	−0.08	0.03	−0.09	0.37	0.06
Diet beverages	0.01	0.53	0.00	0.98	−0.05	0.21	0.01	0.87	0.09	0.59	0.72
Fruit juice	−0.04	<0.001	−0.03	0.58	−0.05	0.05	−0.03	0.67	0.09	0.18	0.51

[1] Mean change (difference between 2018 and 2020) expressed as points for the diet quality score and servings per day for individual food groups.
[2] *p*-values calculated using paired *t*-tests.
[3] *p*-values by race/ethnicity calculated using analysis of variances (ANOVA).
[4] Diet quality score calculated as 0-3 points for higher intake (≤1-3 times/month, 1-6 times/week, once/day, ≥2-3 times/day) for vegetables, fruits, whole grains; 0-3 points for lower intake (≤1-3 times/month, once/week, 2-6 times/week, ≥once/day) of refined grains, red meat, processed meat; 1/0 points for no or <once/month/any consumption of sugar-sweetened beverages, diet beverages, or fruit juices. Change in diet score was calculated by subtracting 2018 score from 2020 score.

Table S4. Mean change in diet quality score (points) and individual food group consumption (servings per day) from before (2018) to during (July/Aug 2020) the COVID-19 pandemic among a subgroup of Cancer Prevention Study-3 cohort participants, stratified by weight change categories during this period (N = 2335).

	Gained ≥ 4.5 kg (N = 332)		Gained 2.25 - <4.5 kg (N = 320)		Lost or gained < 2.25 kg (N = 1,029)		Lost 2.25 - <4.5 kg (N = 321)		Lost ≥ 4.5 kg (N = 333)		*P* by weight change categories [3]
	Mean Change [1]	*P* [2]	Mean Change [1]	*P* [2]	Mean Change [1]	*P* [2]	Mean Change [1]	*P* [2]	Mean Change [1]	*P* [2]	
Diet quality score (points)[4]	−0.40	0.01	−0.18	0.20	0.11	0.21	0.66	<0.0001	1.04	<0.0001	<0.0001
Food groups (servings/day)											
Vegetables	−0.50	<0.0001	−0.24	0.0002	−0.18	<0.0001	−0.09	0.22	−0.10	0.09	<0.0001
Fruits	−0.23	0.001	−0.10	0.07	−0.04	0.30	−0.04	0.51	0.07	0.27	0.02
Whole grains	−0.14	0.004	−0.26	<0.0001	−0.20	<0.0001	−0.08	0.10	−0.11	0.03	0.08
Refined grains	−0.06	0.16	−0.09	0.03	−0.11	<0.0001	−0.07	0.04	−0.22	<0.0001	0.04
Red meat	−0.05	0.08	−0.02	0.37	−0.05	0.0002	−0.05	0.02	−0.06	0.03	0.85
Processed meat	−0.01	0.61	−0.01	0.57	0.00	0.83	−0.05	0.02	−0.04	0.02	0.22
Sugar-sweetened beverages	−0.03	0.49	−0.01	0.69	0.00	0.82	−0.03	0.19	−0.03	0.11	0.63
Diet beverages	0.06	0.13	−0.02	0.47	0.00	0.96	−0.02	0.57	0.01	0.88	0.48
Fruit juice	−0.01	0.59	−0.05	0.02	−0.04	0.0001	0.00	0.99	−0.05	0.01	0.36

[1] Mean change (difference between 2018 and 2020) expressed as points for the diet quality score and servings per day for individual food groups.
[2] *p*-values calculated using paired *t*-tests.
[3] *p*-values by weight change categories calculated using analysis of variances (ANOVA).
[4] Diet quality score calculated as 0-3 points for higher intake (≤1-3 times/month, 1-6 times/week, once/day, ≥2-3 times/day) for vegetables, fruits, whole grains; 0-3 points for lower intake (≤1-3 times/month, once/week, 2-6 times/week, ≥once/day) of refined grains, red meat, processed meat; 1/0 points for no or <once/month/any consumption of sugar-sweetened beverages, diet beverages, or fruit juices. Change in diet score was calculated by subtracting 2018 score from 2020 score.

Table S5. Reported frequency of and mean change in meal sources (times per week) from before (2018) to during (July/Aug 2020) the COVID-19 pandemic among a subgroup of Cancer Prevention Study-3 participants, stratified by sex and race/ethnicity (N = 2335).

		Fast food restaurants		Full-service restaurants		Ready-to-eat meals		Home cooked meals	
	N	Mean Change [1]	*P* [2]	Mean Change [1]	*P* [2]	Mean Change [1]	*P* [2]	Mean Change [1]	*P* [2]
Overall	2335	−0.12	<0.001	−0.47	<0.001	−0.37	<0.001	−0.11	0.002
By sex									
Males	816	−0.15	<0.001	−0.59	<0.001	−0.30	<0.001	−0.17	0.003
Females	1519	−0.11	<0.001	−0.40	<0.001	−0.40	<0.001	−0.08	0.08
By race/ethnicity									
White	1777	−0.11	<0.001	−0.48	<0.001	−0.35	<0.001	−0.15	<0.001
Black	81	−0.33	0.01	−0.18	0.21	−0.59	<0.001	0.09	0.70
Latino/a	298	−0.11	0.03	−0.51	<0.001	−0.41	<0.001	0.04	0.68
Asian/Pacific Islander	88	−0.10	0.26	−0.64	<0.001	−0.25	0.01	0.13	0.45
American Indian/Alaskan Native	44	−0.22	0.12	−0.22	0.14	−0.32	0.03	−0.23	0.37
Other/unknown	47	−0.09	0.59	−0.05	0.70	−0.47	0.02	−0.33	0.14

[1] Mean change (difference between 2018 and 2020) expressed as times per week.
[2] *p*-values calculated using paired *t*-tests.

Supplementary Files

Figure S1. Perceived changes in food group consumption during the COVID-19 pandemic among a subgroup of Cancer Prevention Study-3 participants (N = 2335)

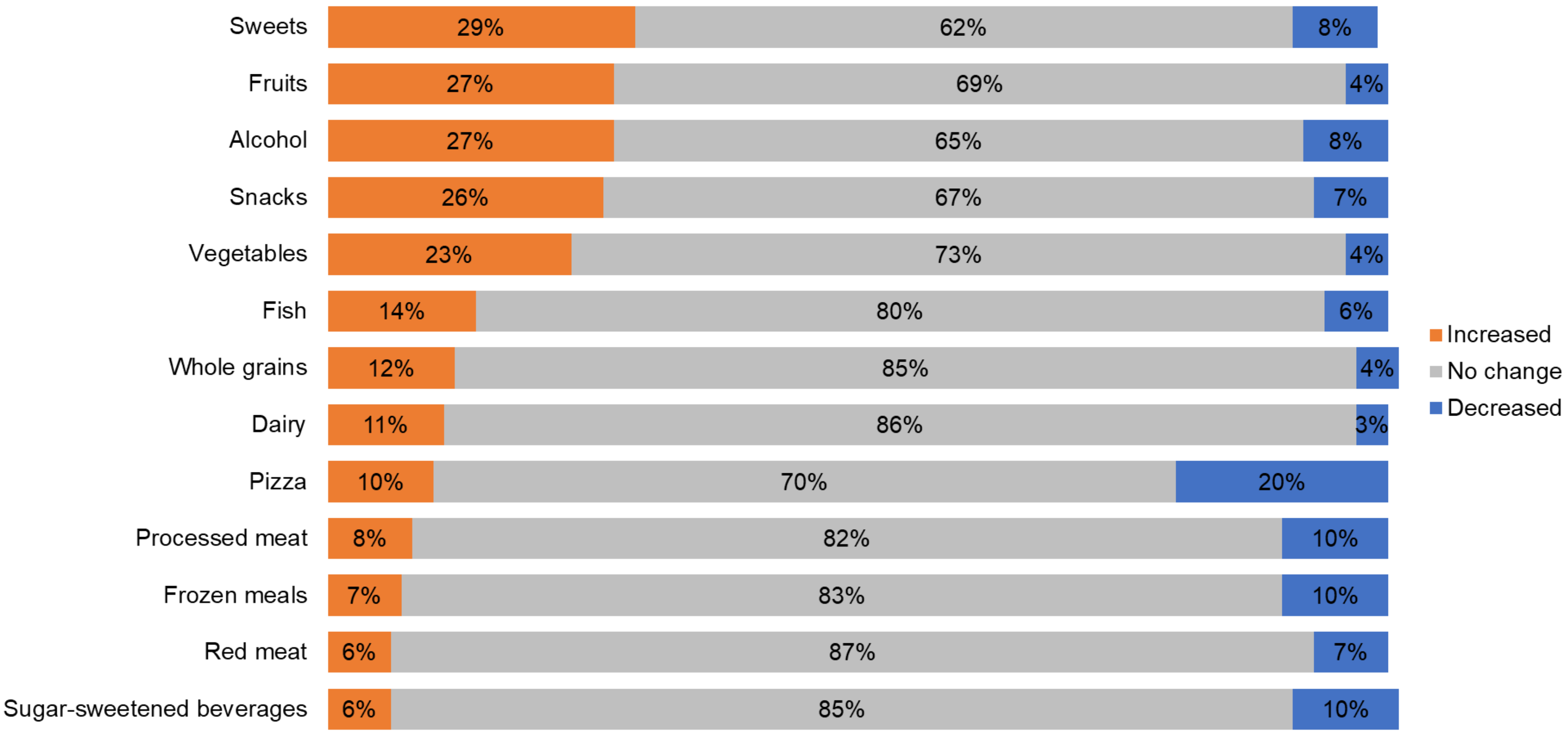

Supplement: Supplementary file 1 [file nutrients-15-00849-s001.zip › Diet and COVID_Supplementary Files proof_Feb 6 2023.pdf]
